# Natural Compounds Targeting the Autophagy Pathway in the Treatment of Colorectal Cancer

**DOI:** 10.3390/ijms24087310

**Published:** 2023-04-15

**Authors:** Yin-Xiao Du, Abdullah Al Mamun, Ai-Ping Lyu, Hong-Jie Zhang

**Affiliations:** School of Chinese Medicine, Hong Kong Baptist University, 7 Baptist University Road, Kowloon Tong, Kowloon, Hong Kong SAR, China; 17482038@life.hkbu.edu.hk (Y.-X.D.); 20481802@life.hkbu.edu.hk (A.A.M.)

**Keywords:** autophagy, natural products, colorectal cancer, lysosome, autophagy inhibitors

## Abstract

Autophagy is a highly conserved intracellular degradation pathway by which misfolded proteins or damaged organelles are delivered in a double-membrane vacuolar vesicle and finally degraded by lysosomes. The risk of colorectal cancer (CRC) is high, and there is growing evidence that autophagy plays a critical role in regulating the initiation and metastasis of CRC; however, whether autophagy promotes or suppresses tumor progression is still controversial. Many natural compounds have been reported to exert anticancer effects or enhance current clinical therapies by modulating autophagy. Here, we discuss recent advancements in the molecular mechanisms of autophagy in regulating CRC. We also highlight the research on natural compounds that are particularly promising autophagy modulators for CRC treatment with clinical evidence. Overall, this review illustrates the importance of autophagy in CRC and provides perspectives for these natural autophagy regulators as new therapeutic candidates for CRC drug development.

## 1. Introduction

Colorectal cancer (CRC) is one of the major causes of cancer-related mortality worldwide. The number of patients diagnosed with CRC annually is over 1.8 million, resulting in about 10% of all cancer-related fatalities [1,2]. The incidence of CRC has been increasing during the last decades. This is attributed to the increasing elder population, unhealthy dietary habits, and improved diagnostics [3]. While laparoscopic surgery is regarded as the primary therapy, much attention has been given to the development of other new treatments [4]. These include radiation therapy, palliative or neoadjuvant chemotherapy, targeted therapy for specific oncogenes, and immune therapy. While some of these have significantly increased average life expectancy and quality of life among CRC patients [5,6,7,8], even more effective, safe, and efficient treatments are sought.

One of the new strategies for treating CRC, as well as other cancers, involves targeting autophagy. Autophagy is a highly conserved intracellular degradation system characterized by the formation of double-membrane vesicles to engulf damaged organelles, toxic proteins, and aggregations of misfolded proteins to be degraded by lysosomes [9,10]. Autophagy plays an indispensable role in maintaining metabolic homeostasis under certain stresses, including nutrient deprivation [11,12]. Increasing evidence suggests that autophagy is also closely related to cancer; however, whether autophagy promotes or suppresses tumor growth is still under debate [13,14]. Nevertheless, it is becoming gradually clear that the loss of autophagy-related genes such as Beclin-1 can promote the earliest stage of tumor formation, and that upregulated autophagy can also assist tumorigenesis in dealing with various stresses such as hypoxia or immune attack [15,16,17]. Thus, targeting autophagy to inhibit tumor formation and metastasis appears to be an alternative strategy for cancer drug development.

In this study, we review the latest knowledge of mechanisms of autophagy in CRC and summarize the information we have on a number of natural compounds reported to target autophagy that have potential value in treating CRC. We hope this paper will inspire further exploration of natural compounds that show potential in regulating autophagy in the treatment of CRC.

## 2. Epidemiology of CRC

CRC is an often fatal disease that is common throughout the world. According to data from the WHO, CRC ranks third among all cancers and is the second leading cause of cancer-related deaths [18]. Nowadays, there are around 900,000 deaths from CRC annually, which represents approximately 10% of all cancer-related deaths [19]. With improved medical service, the average life expectancy of CRC patients has increased dramatically in the last decades. However, mainly due to the prevalence of national screening programs and advanced diagnosis techniques, the incidence of CRC is also rising. 

The exact etiology of CRC is not completely understood. The latest research indicates that both hereditary and environmental risk factors contribute to its development. In epidemiological studies, the majority of CRC cases are sporadic and patients with a negative family history account for 70% of all cases. Of the remaining 30%, it is estimated that 10–20% have CRC in their family, and people are more likely to develop it if their relatives developed this disease at a younger age [20,21]. Again, it is estimated that a subpopulation of around 5–10% of patients inherit CRC. Hereditary CRC can be divided into polyposis syndrome and hereditary non-polyposis colorectal cancer (HNPCC). One of the most common forms of HNPCC is Lynch syndrome, which is highly associated with mutated DNA mismatch-repair genes such as EPCAM, PMS2, and MLHL [22]. These gene mutations impair the normal functions of gene replication and cause the accumulation of DNA mutations which ultimately lead to microsatellite instability (MSI) and increased risk of developing into CRC [23,24]. Some other genetic risk factors, such as mutations of adenomatous polyposis coli (APC) which control the activity of the WNT signaling pathway, and the presence of the mutY DNA glycosylase (MUTYH) gene, are also closely associated with the incidence of CRC [3,25,26]. Besides genetic risk factors, environmental factors including aging, smoking, alcohol consumption, unhealthy physical activity, and obesity may also increase the risk of CRC [19,21]. Even though the actual etiology of CRC is not fully understood, it is widely accepted that most CRC begins with a polyp. According to research, an adenomatous polyp starts from an aberrant crypt in the small intestine, and gradually develops into a neoplastic precursor which evolves into CRC in 10–15 years [27]. Some researchers hypothesize that the origin of the colorectal crypt is the cancer stem cells, or stem-cell-like cells, which form as the result of the dysregulation of the cancer-suppressor gene or oncogene [28,29]. It is crucial to further investigate these cancer-related genes and regulatory mechanisms for the development of possible therapeutic agents and advanced prevention or diagnostic techniques. The earlier the diagnosis, the higher the rate of survival. 

Apart from earlier diagnosis, primarily due to improved diagnostic techniques, the development of better therapies has also contributed to increased survival of CRC patients. Surgery remains the cornerstone of treatment for non-metastasized CRC patients. The outcome of surgery is directly related to the quality of surgery. The usage of laparoscopic resection for primary disease and endoscopic treatment for local, precise malignant polyps dramatically promotes the quality of surgery and relieves the difficulties of convalescence [30,31]. Nevertheless, postoperative magnetic resonance imaging studies indicate that surgical quality can be further improved [32,33]. At the same time, other treatments including the resection of organs affected by metastasis (e.g., liver, lung), neoadjuvant, radiation therapy, and palliative chemotherapy also have beneficial therapeutic effects [34,35,36]. Immune therapy, gene therapy, and systemic treatment also give us new hope and future direction for CRC drug development. Bevacizumab, an anti-VEGF monoclonal antibody, was the first approved biological therapeutic agent for metastatic CRC [37]. PD-1 immune checkpoint blockade antibodies, such as pembrolizumab and nivolumab, have also been approved for CRC patients with dMMR or high MSI [38]. While achievements in anti-CRC drug development and CRC treatments are noteworthy and valuable, the exact pathogenesis of CRC still needs further investigation if we are to cure CRC.

## 3. Autophagy in CRC

### 3.1. Overview of Autophagy

Autophagy is an evolutionally conserved process by which cells maintain metabolic homeostasis. In this process, lysosomes will degrade the misfolded intracellular proteins and damaged or superfluous organelles that are captured by double-membrane vesicles [39,40]. Macroautophagy, microautophagy, and chaperone-mediated autophagy (CMA) are the three distinct forms of autophagy identified in mammalian cells [41]. As the most critical type of autophagy for cellular homeostasis, macroautophagy is featured by forming autophagosomes, which are double-membrane engulfed vesicles. Hereafter, in this review, “autophagy” is used to refer to macroautophagy specifically. Autophagy is mainly triggered by stress conditions such as nutrient deprivation, energy loss, or hypoxia [42]. Since autophagy plays an indispensable role in various physiological processes, e.g., preventing cell damage, regulating cell survival, and recycling damaged organelles, its dysregulation could lead to the development of multiple diseases, including neurodegenerative diseases, cancers, and inflammatory and infectious diseases [43]. Numerous research groups have contributed to elucidating the genetic basis and underlying mechanisms of autophagy from yeast to mammals. It is becoming increasingly clear that autophagy is a multi-step process. Understanding autophagy mechanisms is critical for employing autophagy as a therapeutic target in treating disease.

### 3.2. Molecular Machinery of Autophagy

Substantial research achievements have made progress in revealing the underlying molecular mechanisms of autophagy in the last decades. Our current knowledge is that the process of autophagy consists of at least the following five steps: initiation, vesicle nucleation, maturation, fusion, and degradation. Currently, more than 40 ATG proteins have been identified in yeast; a better understanding of their functions in different stages of autophagy will facilitate an understanding of how autophagy acts as a switch in CRC progression [44]. 

The major trigger for autophagy is the Unc-51-like autophagy activating kinase 1 (ULK1) complex, which mainly comprises ATG13, ATG101, ULK1, and the focal adhesion kinase family protein 200 kDa (FIP200) [45]. The ULK1 complex is continuously inhibited by the mammalian target of rapamycin complex 1(mTORC1), which suppresses the level of autophagy under normal conditions [46]. However, mTORC1 is inhibited by autophagy-inducing conditions such as nutrient starvation, and ULK1 will be autophosphorylated and further phosphorylate other ULK1 components, including ATG13 and FIP200, which ultimately activate the ULK1 complex and autophagy initiation [45,47,48]. The 5′-adenosine monophosphate (AMP)-activated protein kinase (AMPK) can also directly and simultaneously phosphorylate ULK1 and inhibit mTORC1 to induce autophagy under energy-deficient-conditions [49]. While AMPK and mTORC1 are the two main master regulators of the activation of the ULK1 complex, other protein kinases such as tank binding kinase 1 (TBK1) and liver kinase B1 (LKB1) may also activate the formation of autophagosomes [50,51,52]. Once the ULK1 complex is activated, it will be dephosphorylated and localize to the phagophore to activate the Beclin1-VPS34 complex which is required for vesicle nucleation. The VPS34 complex is composed of Beclin1, VPS34 (a class III phosphoinositide3-kinase), VPS15, ATG14, and Beclin1 regulator 1 (AMBRA-1). This complex will bind with WIPIs to form phosphatidylinositol 3-phosphate (PI3P) on the membrane to prepare the phagophore for further elongation [53]. 

After the autophagy signal is initiated, a series of ubiquitin-like conjugation systems will facilitate phagophore expansion and the formation of an enclosed double-membrane vesicle, also known as an autophagosome. ATG12 will conjugate to ATG5 via E1-like ATG7 and E2-like ATG10, then the ATG5–ATG12 conjugate will further associate with ATG16-like protein (ATG16L) to form an ATG5–ATG12–ATG16 complex [54]. This ATG5–ATG12–ATG16 complex is pivotal to another ubiquitin conjugation process in which an ATG8 family protein conjugates to phosphatidyl ethanolamine (PE) which is responsible for autophagosome formation. Microtubule-associated protein 1 light chain 3 (LC3) is one of the most demonstrated members of the ATG8 family of proteins, and it is also regarded as the most accepted marker of autophagosomes in mammals. The firstly synthesized pro-LC3 protein will first be processed by cascade protease ATG4 into the diffuse cytosolic form LC3-I. Upon receiving the autophagy induction signal, ATG5–ATG12 will act as an E3 enzyme to facilitate the insertion of LC3 into the PE membrane-bound group on the emerging phagophore surface where it becomes the lipidated form, LC3-PE, also known as LC3-II [55]. When LC3 is located on the phagophore surface, autophagy cargo receptors with an LC3-interacting region (LIR) can dock with the phagophore surface to facilitate autophagosome formation.

**Figure 1:**
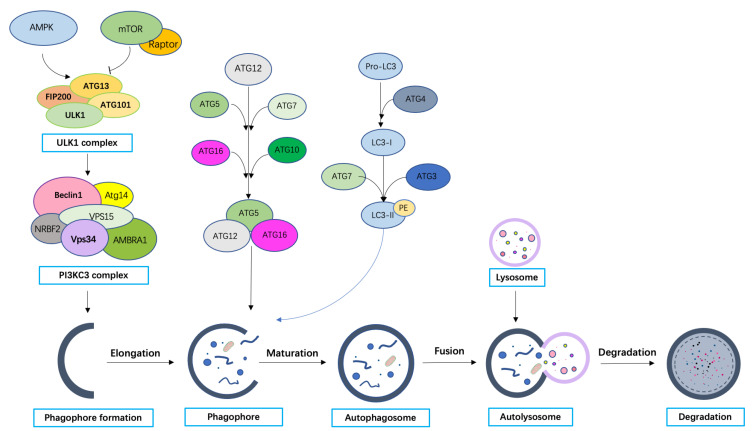
Molecular mechanisms of autophagy in mammalian cells.

Cargo proteins, including SQSTM1(p62), a neighbor of BRCA1 gene 1 protein (NBR1), and other selective autophagy cargo receptors, such as nuclear protein 52 kDa(NDP52) and optineurin(OPTN), will recruit the misfolded protein aggregations or damaged organelles to the phagophore for subsequent autophagic degradation [56,57,58,59,60]. Additionally, other lipid sources will be shuttled from various membranes, such as Golgi networks, endosomes, or plasma membranes, to phagophore by ATG9 where they will help close the membrane, thereby trapping the cargo. This becomes the completed double-membrane autophagosome [61,62,63]. The established autophagosome will become mature, meaning that most ATG proteins will be dissociated (mostly by removing ATG8 and PI3P from the surface of autophagosomes) so that they can fuse with a lysosome [64]. Once the autophagosome successfully fuses with the lysosome, the cytosolic macromolecules, aggregated protein, and damaged organelles will be degraded inside the autolysosome (Figure 1) by acid lysosomal hydrolases [65]. 

### 3.3. Dual Role of Autophagy in CRC Development

Autophagy plays an indispensable role in the development of CRC. At different stages or in different subtypes of cancer, it either induces or inhibits cancer growth. On the one hand, autophagy’s recycling of damaged cellular materials and accumulated oncogenesis components provides an alternative energy source to support cancer cell survival. On the other hand, autophagy in cells under stress or certain microenvironments could diminish cancer cell proliferation. It is generally believed that autophagy acts as a tumor-suppressive factor at the early stage of tumorigenesis and turns into a tumor-promoting factor once the cancer is established [66]. While the exact role of autophagy in tumorigenesis and metastasis remains controversial, increasing clinical evidence and molecular experiment results suggest that inhibiting autophagy increases the effect of anticancer therapies in advanced cancer patients. Understanding the dual roles of autophagy in CRC is crucial for the further development of effective anticancer drugs.

#### 3.3.1. Tumor Inhibiting Function of Autophagy

Autophagy plays an important role in removing the damaged structures or harmful chemicals in normal cells, and its impairment will lead to the aggregation of mutations and other oncogenesis components, promoting tumor development in the earliest stages of cancer. In support of this point of view, numerous researchers have shown that modulating autophagy-associated protein to manipulate autophagy affects the progression and proliferation of CRC.

BECN1(encoding Beclin-1 protein) is a mammalian ortholog of the yeast autophagy-related gene 6 (Atg6); it participates in the initiation of the autophagy pathway and cell death [67]. BECN1 has been generally identified as a tumor-suppressing gene in the last decades because decreased BECN1 expression level is widely observed in various types of cancer, including breast cancer, glioblastoma multiforme, and ovarian and prostate cancer [68,69,70]. Ectopic overexpression of BECN1 suppresses the proliferation, survival, and migration of different types of cancer cells [71,72]. Moreover, BECN1 deficiency in embryoid bodies accelerates embryonic cell death, and spontaneous tumorigenesis is promoted in heterozygous BECN1-disrupted mice. This evidence further supports the tumor inhibition function of BENC1 [73,74]. 

Conversely, the latest research also found upregulated BECN1 expression in the majority of 363 colorectal and gastric cancer patient samples compared with individual colon and stomach mucosa samples from healthy individuals [75]. However, whether the expression level of BECN1 is up- or down-regulated in colorectal cancer cells remains controversial [76]. BECN1 could exhibit its tumor-suppressive character in CRC patients with BECN1 defect, which was supported by the evidence that activation of autophagy through overexpressing BECN1 showed a therapeutic effect and inhibited CRC growth [77]. On the other hand, the higher expression of BECN1 may be correlated with tumor hypoxia and may facilitate patient survival, suggesting that BECN1 may be a reliable marker for prognosing most patients with CRC [75]. Another autophagy-related protein, UVRAG, that interacts with Beclin-1 to initiate autophagy, has also shown a tumor-suppressive role in CRC. Monoallelic loss or mutation of UVRAG has been frequently reported in colon, gastric, or breast cancers, and mutations of UVRAG attenuate autophagy levels and promote tumorigenesis in colorectal cancer [78,79].

#### 3.3.2. Tumor Promoting Function of Autophagy

Even though mounting evidence indicates that autophagy might play an inhibiting role in the early stage of CRC, there is also evidence that autophagy is beneficial for tumorigenesis in developed cancer. Autophagy can also be activated by some oncogenic risk factors or tumor microenvironment stresses such as hypoxia. Autophagy may provide supplementary energy support or recycling to sustain the tumor cell survival and invasion [80]. For instance, mutations of the Kristen rat sarcoma virus (KRAS) and neuroblastoma RAS viral oncogene (NRAS) are encountered in approximately 44.7% and 7.5%, respectively, of all colorectal carcinoma cases [81]. It has been demonstrated that mutations of the RAS family gene have a contributory role in the development and progression of colorectal adenomas and that the autophagy level is highly elevated in these RAS-driven cancer cells [82,83]. RAS activation promotes the reliance of tumor cells on increased autophagy to maintain energy production and metabolism under nutrient deprivation. Inhibition of autophagy will reverse the development of the carcinoma and lead to tumor regression [84,85,86]. Suppressing autophagy by deleting ATG5 or ATG7 will prevent malignant tumor formation and slow the development of the tumor [83,87,88]. These studies showed that activated autophagy is also prevalent in developed cancer, and inhibiting autophagy can sometimes display beneficial effects in non-Ras driven tumoral cancer cells.

While autophagy has been identified as a tumor-promoting factor under some oncogenic genes such as KRAS or NRAS in CRC, autophagy can also act as an adaptive mechanism to various stresses that support cancer cell survival and growth in spontaneous CRC. Tumors usually possess a high proliferation rate and outgrow their existing blood supply, resulting in microenvironmental stresses such as hypoxia and nutrient shortage. Even though angiogenesis in the tumor can slightly relieve these stresses, the lack of ATP and metabolites needs increasing autophagy to maintain cancer cell viability [89]. HMGB1 (high mobility group box 1) is an abundant protein that regulates inflammation, proliferation, and metastasis in different cancer models. The continuously upregulated mucosa concentration of HMGB1 in the CRC model will induce autophagy to accelerate the aged protein or damaged organelles recycling for energy production and amino acid supply [90,91]. The lower ATP level driven by hypoxia will also activate AMPK, the master energy regulator, to induce autophagy to support tumor survival [92]. Many studies have reported that inhibition of autophagy by autophagy modulators could induce cancer cell apoptosis and prolong the life of cancer mouse models [86,93]. Genetic depletion of FIP200 also inhibits the initiation of autophagy and suppresses cancer cell growth in colorectal and breast cancer models [94,95]. Furthermore, the selective organelle autophagy-dependent degradation of unnecessary mitochondria (also known as mitophagy) is also activated in solid tumors to provide extra nutrients and expedite glycolysis [96]. Therefore, upregulated autophagy in developed tumors increases the microenvironmental stress tolerance and provides nutrients and energy, which further supports tumor cell survival and tumorigenesis progression. It is generally believed that autophagy is a tumor-promoting factor in developed cancer, and inhibition of autophagy is considered a promising pathway for advanced CRC drug discovery. 

Another autophagy-related gene BECN1, alteration or deletion of other ATG genes such as ATG5 or ATG7 has been demonstrated to enhance CRC tumorigenesis. Recent research reported intestinal adenoma and augments interferon-gamma’s therapeutic effects were increased when systemic heterozygous removal of ATG5 occurs in ApcMin/+ mice, revealing that autophagy inhibition through interfering with ATG5 function could be an efficient strategy for preventing and treating CRC [97]. Moreover, recent research has also reported the loss of ATG5 gene and downregulated ATG5 expression level in CRC patients [98,99]. Employing the microRNA miR-183-5p to target ATG5 also enhances the radioresistance of CRC, and inhibiting autophagy through interfering with ATG5 may trigger cancer cell apoptosis by p53 activation, ER stress, and the UPR pathway. This indicates that the autophagic pathway may suppress tumor progression and facilitate the chemosensitization of CRC cells [87,100]. Besides ATG5, other ATG genes such as ATG7 are also involved in CRC development. Intestinal dysbiosis is caused by a deficiency of Atg7, which in turn inhibits tumor development via an immunological response controlled by the microbiome [101]. Taken together, these results demonstrate that disruption of autophagy-related genes promotes the tumor-suppressing function of autophagy in the early stages of CRC formation.

#### 3.3.3. Autophagy and Chemotherapy Resistance in CRC

Drug resistance occurs in many forms of cancer, including CRC, and is often the result of increased autophagy, which protects cancer cells from the effects of chemotherapy [102,103]. Although the precise mechanism by which autophagy contributes to chemo-drug resistance is not fully understood, the therapeutic effect of a combination of chemotherapy with autophagy inhibitors is supported by abundant evidence. Some researchers report that autophagy participates in the cell death process in cancer which further sensitizes the chemotherapy effect. Autophagy may impact drug resistance through the following pathways: activation of pro-apoptotic factors, inactivation of anti-apoptotic effectors, and enhancement of cancer cell apoptosis signals [102]. Currently, there is increasing evidence that employing small interfering RNA (siRNA) to silence ATG genes in order to inhibit autophagy could sensitize resistant cancer cells to chemotherapy. The autophagy inhibitor 3-Methyladenine (3-MA) that blocks autophagosome formation via type III phosphatidylinositol 3-kinases (PI-3K) enhanced the effect of 5-fluorouracil (5-FU)-induced apoptosis in colon cancer cells [104]. Other autophagy inhibitors, such as chloroquine or hydroxychloroquine that impair the fusion of lysosome and autophagosome, also potentiate the anticancer efficacy of 5-FU on CRC cells [105,106]. Knocking down ATG proteins such as ATG7 is another strategy to be explored to augment the chemotherapy resistance in CRC [88]. In a Phase II clinical trial, the combination of FOLOX (a chemo therapy for CRC, FOL = Leucovorin Calcium (Folinic Acid), F = Fluorouracil, OX = Oxaliplatin) with autophagy inhibitor hydroxychloroquine enhanced cancer cell death, resulting in a higher objective response rate (ORR) in the patients with metastatic CRC (NCT01006369). Isoliquiritigenin is an anti-cell cycle chemotherapeutic agent that can suppress the growth of various tumors by inducing G2 and M phase arrest. At the same time, a classic autophagy inhibitor 3-MA enhances isoliquiritigenin’s anticancer effect by regulating cell cycle arrest [107]. Conversely, other studies have shown that the 5-FU therapeutic response is regulated by the balance between apoptosis and autophagy in cancer cells, which is regulated by mitogen-activated protein kinase 14 (MAPK14)/p38 activation [103]. Chloroquine was also reported to suppress the (MAPK14)/p38α- mediated cell protective autophagy and cytotoxic effects of 5-FU in CRC [108,109]. Although the exact underlying mechanism of how autophagy regulates drug resistance remains elusive, many clinical trials of the combination of autophagy pharmacological modulators with chemotherapy or radiation therapy are in progress, which may provide a novel therapeutic strategy for treating CRC.

#### 3.3.4. Autophagy and Gut Microbiota in CRC

The population of gut microbes has been identified as a key player in cancer pathology alongside other genetic and environmental risk factors that contribute to the development of CRC disease during the past decade. Changes in gut microbiota have been linked to CRC development in multiple animal studies [110,111]. Early research in 1967 found that glucoside cycasin, a typical hepatotoxin and carcinogen in conventional rats, failed to induce tumors in germ-free rats. This was the first evidence that intestinal microorganisms played a role in CRC development [112]. Subsequent researchers found that the presence of specific species of intestinal microbiota, e.g., Escherichia coli and Enterococcus faecium, correlated with increased aberrant crypt foci and colon carcinogenesis in 1,2-dimethylhydrazine-induced colon rodent models [113]. Transferring fecal samples from CRC patients promoted tumorigenesis and induced colon neoplasias in germ-free mice and conventional mice treated with azoxymethane [114]. Using advanced sequencing technologies such as human shotgun or 16S ribosomal RNA(rRNA) to compare the gut microbiota changes in CRC, the latest metagenomic or metataxonomic studies revealed a global compositional alteration of gut microorganisms between healthy individuals and patients with CRC [110,115,116]. Some researchers hypothesize that tumor-infiltrating microbes in colonic mucosa will persistently induce innate colonic lymphoid cells to produce inflammatory cytokines such as IL22 or IL17, further promoting cancer cell proliferation and genotoxic substance accumulation [117,118,119,120]. 

#### 3.3.5. Autophagy and Immunotherapy in CRC

Immunotherapy is a kind of treatment that targets tumor-specific antigens to boost the immune system to combat various types of cancer. In view of the adverse effects of chemotherapy and radiotherapy, immunotherapy is being explored as a safer, possibly more effective clinical approach to treating cancer. In the context of CRC, several immunotherapies have been employed, including checkpoint inhibitors, autologous vaccines, dendritic cell vaccines, peptide-based vaccines, adoptive cell transfer, and toll-like receptor (TLR) agonists [7,121,122]. However, since autophagy is a potential target for CRC drug development and a pivotal regulator for cellular protein homeostasis, several pieces of clinical and experimental evidence suggest that a combination with immunotherapy and autophagy inhibition is beneficial for the curative outcome of CRC. 

The exact role of autophagy in the anti-tumor immune response is not clear. One possibility is that autophagy facilitates the colorectal tumor-associate antigen (TAAs)-presenting process and exaggerates the specific immune epitope, such as VEGF (vascular endothelial growth factor), EGFR (epidermal growth factor receptor), TP53, or KRAS, which further boosts the peptide vaccines that target on these potential therapy antigens [123,124]. In some advanced mouse models of metastatic liver tumors, chloroquine (autophagy inhibitor) could reduce tumor development and improve the effectiveness of immunotherapy with interleukin-2 while limiting toxicity [125,126]. Chloroquine also switches the tumor-associated macrophages from M2 to tumor-killing M1 phenotype, which further changes the tumor microenvironment to restrain the immunosuppressive infiltration of myeloid and Treg cells, ultimately supporting antitumor T-cell immunity [127]. Both autophagy inhibition by ATG3 and ATG7 knockdown and pharmacological inhibition with chloroquine energize the immunotherapy anti-PD1 and anti-CTLA4 antibodies and eventually lead to tumor regression and enhanced immune response in pancreatic cancer by regulating MHC-1 degradation through autophagy [128].

## 4. Natural Compounds That Modulate Autophagy as Potential CRC Treatments

**Figure 2:**
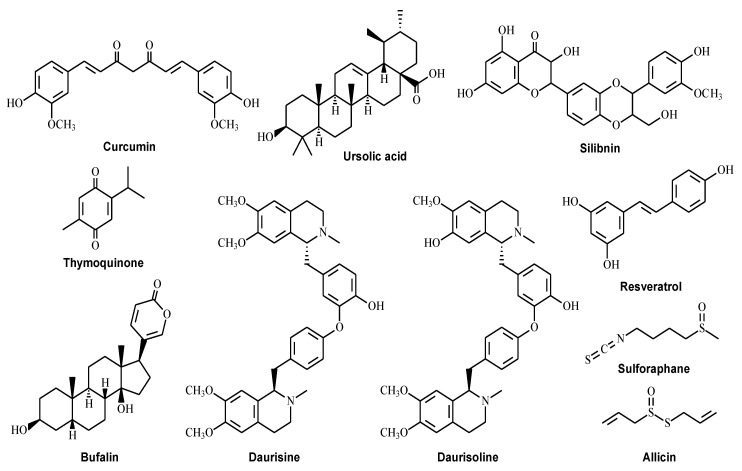
Structures of the natural compounds for the treatment of colorectal cancer by targeting the autophagy pathway.

Autophagy is a highly conserved self-degradation process which plays a significant role in controlling overall cell metabolism, senescence, and stress responses. However, the dysregulation of autophagy contributes to tumorigenesis, and targeting autophagy is also regarded as a potential and effective cancer therapeutic strategy. Earlier research on pharmacological small molecules of natural products that modulate autophagy mainly focused on a few druggable targets, including autophagy initiation, autophagosome formation, and the autolysosome fusion process. Chloroquine or hydroxychloroquine is a 4-aminoquioline drug that has been widely accepted as an autophagic inhibitor to inhibit lysosome acidification and block autophagosome and lysosome fusion [129]. Chloroquine significantly exhibited anti-tumor properties since it enhanced the cytotoxicity effect of temsirolimus and alleviated the autophagy-dependent drug resistance to 5-FU [130]. Another autophagy inhibitor, 3-MA, inhibited autophagy at the early stage by suppressing autophagosome formation. The autophagy inhibitor 3-MA also showed the anti-tumor effect that enlarged the apoptosis-inducing effect of 5-FU in colon cancer [104]. Besides the drugs that directly target the process from autophagosome initiation to autolysosome formation, acting on upstream signaling pathways including mTOR, AMPK, and PI3K is also available in regulating autophagy in various cancer treatments [131]. Rapamycin is a commonly used mTOR inhibitor to induce autophagy upstream of the ULK1 pathway. It can also induce colon cancer cell apoptosis, providing promising therapeutic benefits for patients at high risk of developing CRC [132,133]. More natural compounds have been identified as potential autophagy regulators in the last decades [134]. These small natural molecules may possess lower toxicity and a greater ability to regulate autophagy than conventional drugs. Here we summarize (Table 1) information on some well-known natural compounds (Figure 2) that have been reported to exhibit anti-tumor effects through modulating autophagy. 

### 4.1. Curcumin

Curcumin is a polyphenolic compound that is isolated from turmeric, which is the rhizome of Curcuma longa L., a member of the ginger family. Curcumin has various, medically relevant bioactivities, including anti-inflammatory, antioxidant, cardioprotective, hepatoprotective, anti-aging, and anti-carcinogenic properties [135,136,137]. It has been found that curcumin induces autophagic cancer death, and that it appears to target multiple molecules, such as AKT, mTOR, and activator protein 1(AP-1) [138,139,140]. Studies show that treating malignant gliomas with 40 μM curcumin could promote autophagy and reduce tumor growth by activating the ERK1/2 pathway or blocking the AKT/mTOR pathway [141]. Additionally, curcumin was found to inhibit the growth of human colon cancer cell lines HT-29 and HCT-15 in dose-dependent manners and it could arrest the accumulation of cancer cells in the G2/M phase of the cell cycle [142]. Zhu et al. found that curcumin suppressed the proliferation and induced autophagy in HCT116 and SW620 human colon cancer cells by upregulating the expression of LC3-II and downregulating the expression of P62 and the yes-associated protein (YAP) [143]. Another research group has reported that curcumin-induced autophagic cell death was activated through increased production of reactive oxygen species (ROS) in HCT116 human CRC cells, resulting in the accumulation of LC3-II and autophagosome development [144]. Curcumin has been shown to inhibit CRC proliferation by enhancing autophagic cell death. It also synergizes with 5-FU to enhance CRC cell apoptosis and inhibit tumor growth in xenograft mice by impairing the AMPK/ULK1 pathway [145]. The latest paper also suggested that curcumin therapy could increase autophagic flux in HCT116 cells and mouse embryonic fibroblasts (MEFs) by inhibiting mTOR and activating transcription factor EB (TFEB) [146].

Analogs of curcumin have also been shown to inhibit carcinoma progression by inducing autophagy-mediated cell death. A new water-soluble curcumin analog, 3,5-bis(2-hydroxybenzylidene)tetrahydro-4H-pyran-4-one glutathione conjugate [EF25-(GSH)2], induced autophagy at both low (5 μM) and high dosage (10 μM), and the longer treatment of the compound would induce more cell death through both an apoptosis-dependent and a non-apoptotic mechanism in a human hepatic cell line HL-7702 [147]. Another curcumin derivative, bis-dehydroxycurcumin (bDHC), activated mitochondrial-associated cell death with Bcl-2 reduction and activation of ER stress in the HCT116 cell line [148]. A recent study by Mao et al. found that curcumin inhibited LGR5(+) colorectal cancer stem cells, possibly by activating autophagy and suppressing the oncogenic TFAP2A-mediated ECM pathway [149]. Another study demonstrated that curcumin significantly downregulated the expression of DCLK1/Lgr5/ALDHA1 (stem cell markers) and Nanog (pluripotent marker), accompanied by a significant decrease in levels of total β-catenin and activated NF-κBp65^s276^ and a 3-fold increase in levels of LC3-II and activated caspase-3 in HCT-116 xenografts [150]. In summary, curcumin and its analogs may trigger autophagic cell death and augment the cytotoxicity of chemotherapeutic agents to inhibit CRC progression.

### 4.2. Ursolic Acid

Ursolic acid is a natural pentacyclic triterpenoid with a carboxylic acid functional group that is present in many plants. For pharmaceutical use and experimentation, it is mainly derived from the leaves of Eriobotrya japonica, Prunella vulgaris L., and Ilex rotunda Thunb. An increasing amount of evidence indicates that ursolic acid possesses anti-inflammatory, anti-hyperlipidemic, and anti-cancer properties, which has attracted interest in using it in advanced solid tumor therapy in clinical trials [151,152,153]. Ursolic acid induces autophagy activated through the CaMKK-AMPK-mTOR pathway, and also activates autophagy by stimulating ER stress caused by enlarged Ca^2+^ release in U87MG cells [154]. Ursolic acid can also induce cancer cell autophagy and caspase-independent apoptosis, and when it was co-administered with 5-FU, 5-FU’s effect on HCT15 CRC cells could be further enhanced. In an in vitro study, ursolic acid activated autophagy with the accumulation of LC3 and p62 and activated caspase-independent cancer cell apoptosis through the JNK pathway. The in vivo study also showed that ursolic acid suppresses tumor development in murine xenografted with HCT15 cells [155]. In addition, employing small interfering RNA(siRNA) to silence the ATG5 gene significantly weakened the ursolic acid-induced LC3-II increase and cytotoxicity of TC-1 cervical cancer cells, which suggests that modulating ATG5 may amplify the anti-tumor effect of ursolic acid [156]. Another paper found that ursolic acid induced breast cancer cell autophagy and apoptosis through PI3K/AKT-controlled GSK3β and elevated caspase-3 associated with NF-κB signaling pathways [157]. In contrast, other papers report that ursolic acid induces PC-12 cell death while impairing the downstream steps in autophagy [158]. Nevertheless, ursolic acid is widely accepted as a potent anti-tumor agent, and it is considered to induce cancer cell death through mediating autophagy.

### 4.3. Silibinin

Silibinin is a natural polyphenolic flavonoid mainly derived from *Silybum marianum* (L.) Gaertn. It possesses anticancer, antioxidant, and anti-inflammatory properties and has commonly been used as a herbal supplement for liver disorder treatment [159]. Autophagy is activated by silibinin via a BNIP3 mediated oxidative stress or the mTOR inhibition to contribute to cancer cell apoptosis [160,161,162]. Silibinin at different dosages (50–200 µM) significantly suppressed the growth and progression of human CRC; it appears to work by interfering with NF-κB signaling activation [163]. Silibinin strongly suppressed tumor necrosis factor α-induced NF-κB signaling. It exhibited a promising tumor inhibition effect on oral silibinin feeding SW480 (COX-2 negative) and LoVo (COX-2 positive) tumor xenografts nude mice. There is evidence that a 200 mg/kg/d dose of silibinin can inhibit tumor growth and cancer cell proliferation in HT-29 xenografted nude mice through down-regulation of ERK1/2 and the phosphorylation of AKT [164]. The proliferation of LoVo human CRC cells was greatly inhibited by silibinin, and apoptosis was triggered as evidenced by elevated levels of cleaved caspases (3 and 9) and cleaved poly (ADP-ribose) polymerase. The strong G1 phase and a slight G2-M-phase cell cycle arrest caused by high-dosage silibinin treatment further enhance the tumor inhibition efficacy in advanced human CRC. Raina et al. [165] demonstrated that silibinin induced autophagy in colorectal cancer, as evidenced by a considerable time-dependent increase in the ratio of LC3-II to LC3-I in SW480 cells. Silibinin was also found to reduce sequestosome 1 (SQSTM1) protein levels while simultaneously increasing SQSTM1 mRNA levels [165]; SQSTM1 is a specific substrate of autophagy, and its protein levels are reduced during starvation-induced autophagy [166]. They also examined the expression of SQSTM1 in silibinin-treated CRC xenograft tissues for investigating the involvement of silibinin-induced autophagy in colon cancer in vivo [165]. According to the findings, silibinin feeding reduced SQSTM1 immunoreactivity by about 28% in SW480 tumor tissue, suggesting that the treated tissues had been stimulated to undergo starvation-induced autophagy [165].

### 4.4. Thymoquinone

Thymoquinone is a natural hepatoprotective, anti-inflammatory and anticancer agent isolated from Nigella sativa L. or black cumin [167]. Thymoquinone therapy significantly decreased tumor cell invasion and slowed down tumor growth in two mouse colorectal cancer models (1, 2-dimethyl hydrazine induced tumor and xenograft) via activating autophagic and apoptotic pathways [168]. Thymoquinone also augmented chemotherapy cytotoxicity against 5-FU-resistant colorectal cancer cells which further induced DNA damage and apoptotic cancer cell death [169]. Similarly, other research has also shown that combing thymoquinone and 5-FU or using a novel 5-FU/thymoquinone hybrid potentiated 5-FU effect through regulating WNT/β-catenin and PI3K/AKT signaling pathway to target cancer stem cells of CRC or the early stages of colorectal carcinogenesis in rats [170,171]. Another paper also suggested that thymoquinone chemosensitized colon cancer cells by inhibiting NF-κB signaling to downregulate the chemosensitization-related VEGF, c-Myc, and Bcl-2 expression levels [172]. Furthermore, thymoquinone has also been reported to increase mitochondrial outer membrane permeability, thereby initiating autophagic cell death in an irinotecan-resistant (CPT-11-R) LoVo colon cancer cell line. In this case, the cancer cell death was also reversed by the presence of JNK and p38 MAPK inhibitors [173]. In contrast, another research indicated that thymoquinone suppresses autophagy and activates cathepsin-mediated cell death in glioblastoma cells [174]. Exposure to thymoquinone caused an increase and accumulation of the microtubule-associated protein light chain 3-II (LC3-II) and autophagosome cargo protein p62 in a dose-dependent manner (from 10 to 40 μM), which strongly suggested that thymoquinone inhibits the late stage of autophagy. Thymoquinone is a promising candidate for use as an anti-CRC agent, whereas the underlying mechanisms of how thymoquinone regulates autophagy still need further investigation.

### 4.5. Dauricine and Daurisoline

Both dauricine and daurisoline are natural isoquinoline alkaloids extracted from the traditional Chinese medicine Rhizoma Menispermi, the dried root of Menispermum dauricum DC. Both dauricine and daurisoline have been identified as potent autophagy inhibitors through a high throughput screening. Dauricine and daurisoline impaired autophagy at the autophagosome maturation stage after the autophagic substrate p62 accumulation and the increased ratio of GFP-LC3/RFP-LC3 [175]. Dauricine is reported to suppress the proliferation and invasion of colon cancer by inhibiting the NF-κB pathway and inducing cancer cell apoptosis in a dose- and time-dependent manner [176]. Dauricine is regarded as a small-molecule autophagy enhancer that stimulates the AMPK-mTOR-dependent autophagy pathway to autophagic cell death in apoptosis-defective cells [177]. In one study, dauricine inhibited the viability of renal cell carcinoma cells and caused cell cycle arrest at the G0/G1 phase by PI3K/Akt signaling pathway inhibition [178]. Another novel analog of dauricine, N-desmethyldauricine, is able to induce autophagic cancer cell death by mobilizing calcium release from SERCA to activate the calmodulin-dependent protein kinase kinase β (CaMKKβ)-AMPK-mTOR signaling cascade [179,180].

### 4.6. Bufalin

Bufalin is a polyhydroxysteroid isolated from the skin or the parotid venom glands of various toad species; in Chinese medicine, it is known as Venenum Bufonis. Befalin has been widely accepted as a bioactive cardiac glycoside or anticancer agent. In addition to its anticancer properties, recent studies have indicated that bufalin could inhibit CRC cell growth and prevent tumor formation by modulating autophagy. In the latest study, bufalin effectively prevented colon tumorigenesis and suppressed the expression levels of pro-inflammatory mediators in two mouse models of CRC [181]. The administration of 0.5 mg/kg bufalin significantly decreased colon tumor incidence in both colitis-associated colon carcinogenesis induced by azoxymethane/dextran sulfate sodium treatment in BALB/c mice and Apc germline mutant mice. Bufalin may inhibit CRC proliferation and downregulate a series of colorectal inflammatory cytokines by suppressing NF-κB and PI3K/AKT signaling pathways [181]. One research group demonstrated that bufalin induced autophagy and caused autophagy-related cell death in HT-29 and Caco-2 colon cancer cells through reactive oxygen species (ROS) generation and JNK activation [182]. It has also been demonstrated that bufalin induces autophagy and apoptosis by inhibiting the phosphorylation of Akt and mTOR, while phosphorylated ERK1/2 was enhanced by bufalin in gastric cancer cells [183]. In bufalin-treated colon cancer cells and gastric cancer cell lines, increased cleaved caspase-3 or cleaved PARP were reported, followed by accumulation of the autophagy marker LC3-II and decrease in p62 level, indicating the autophagy pathway had been activated [182,183]. Bufalin has also exhibited an anticancer effect in HCT116 human CRC cells and an orthotopic xenograft mice model. In the animal study, phosphorylated AKT and Bcl-xL were observed to be downregulated to initiate autophagy-related apoptosis [184]. Another line of research has found that combined treatment of bufalin and 5-FU could induce higher levels of apoptosis than 5-FU alone and prevent cancer cell proliferation in CRC cells by the mitochondrial apoptotic pathway [185].

### 4.7. Resveratrol

Resveratrol is a natural stilbenoid present in the Polygonum cuspidatum, the skin of grapes, red wine, berries, peanuts, and other common foods [186]. Resveratrol has been identified as a novel and promising drug candidate with diverse pharmacological activities, including antimicrobial, antioxidant, and anti-inflammatory activities; its cancer-preventive and therapeutic properties have been widely studied in different human cancers over the last decades [186,187,188]. In DLD1 and HCT15 colon cancer cell lines, resveratrol inhibited cancer cell proliferation correlated with the induction of apoptosis and G1 phase cell cycle arrest without affecting normal colon epithelial cells [189]. In one study, resveratrol significantly inhibited the WNT/β-catenin signaling pathway by regulating metastasis associated with lung adenocarcinoma transcript 1(MALAT1) in LoVo and HCT116 CRC cell lines at the dosage of 50μM, ultimately suppressing CRC invasion and metastasis [190]. A clinical trial of resveratrol in CRC found that eight daily doses of resveratrol at 0.5 or 1.0 g before surgical resection slightly reduced cell proliferation in twenty patients, which revealed that the use of resveratrol as a potential chemopreventive agent against CRC is clinically safe [191]. The relationship between resveratrol and autophagy activation has been widely studied in various cancers, and the underlying molecular mechanisms have also been illustrated [192,193,194]. Miki H. et al. showed that resveratrol induced a dose- and time-dependent activation of autophagy and apoptosis in HT-29 and COLO 201 human colon cancer cells. This was shown by electron microscopy and by the elevation of LC3II level in immunoblotting tests [195]. Resveratrol increased the intracellular ROS level to induce autophagy, evidenced by that N-acetyl cysteine induced ROS inhibition diminished the autophagic-related apoptosis effect in CRC. Similarly, it was also reported that resveratrol activates autophagy through inhibition of the mTOR activity by its direct docking on the ATP-binding site of mTOR in MCF-7 cancer cells. mTOR inhibition is essential for the autophagy initiation and senescence, resulting in the suppressed viability of MCF-7 cells [196]. According to another study, resveratrol causes autophagy-mediated cell death in DU145 and PC3 cells via reducing ER calcium accumulation and store-operated calcium entry [197]. The study further revealed that resveratrol inhibited stromal interaction molecule 1 (STIM1) expression level and AKT/mTOR pathway to trigger autophagic flux [197]. Resveratrol also exhibited the ability to regulate chemotherapy resistance in SW480 and LoVo cell lines through sensitizing CD133+ cancer stem cell apoptosis. It appears that the combination treatment of 5-FU and resveratrol can also upregulate CRC apoptosis by regulating the BAX gene [198]. A study by Hong et al. [199] found that trans-scirpusin A (TSA), a resveratrol oligomer, induced autophagy in Her2/CT26 cells by increasing the amount of the mammalian autophagy protein LC3 puncta and an increase in the conversion of LC3-I to LC3-II. In addition, TSA induced p-AMPK and inhibited mTORC1 activity as measured by the expression level of p-p70S6K, indicating that TSA-induced AMPK activation and suppression of the mTORC1 pathway may be related to autophagy induction.

### 4.8. Sulforaphane

Sulforaphane is a natural aliphatic isothiocyanate compound widely found in cruciferous vegetables such as broccoli, cabbage, and kale. Sulforaphane has shown significant pharmacological activities, including neuroprotective and anticancer effects [200,201]. It was first developed as a phytoantioxidant to protect cells from oxidative stress. Sulforaphane has shown anticancer activities in various types of cancer including breast cancer, prostate cancer, and CRC. Moreover, a phase II clinical trial to evaluate the effect of sulforaphane on breast, bladder, and prostate cancer was carried out in the last decade [201]. In the field of CRC study, sulforaphane at concentrations of 10 to 100 μM inhibited HT-29 colon cancer cell proliferation [202]. A similar result was reported in a later research paper. In the study, sulforaphane reduced the growth of three unrelated CRC cell lines (SW480, DLD1, and HCT116) by modulating the Wnt/β-catenin signaling pathway [203]. However, the opposite effects were reported for the compound in another study, which found that sulforaphane promoted p53-WT HCT116 cancer cell proliferation in a biphasic manner via Nrf2 activation and p53-dependent flux [204]. Sulforaphane’s ability to modulate autophagy has further been investigated in various cancer cells. In PC-3 and LNCaP human prostate cancer cells, sulforaphane increased the formation of autophagosomes and expression of LC3, which suggests the induction of autophagy [205]. In prostate cancer cells, it has been shown that sulforaphane-activated autophagy inhibited release of cytochrome c and apoptosis. In the study carried out in human CRC HT-29 and SW480 cells, sulforaphane limited carcinogenesis and upregulated the UDP glucuronosyltransferase 1A (UGT1A) level through the ERK/Nrf2 signaling pathway [206]. In neuronal cells, sulforaphane also has a similar autophagy induction effect through ERK activation [207]. On the other hand, the suppression of autophagy facilitated sulforaphane-induced apoptosis [208]. Nishikawa et al. [93,209] showed that a specific autophagy inhibitor (3-MA) increased the proapoptotic effect of sulforaphane in WiDr colon cancer cells, which was reliant on caspase activation and cytochrome c release into the cytosol. These findings support the use of the chemopreventive agent sulforaphane as a multipotential anti-colon cancer agent in conjunction with autophagy inhibitors.

### 4.9. Allicin

Allicin is an organosulfur compound mainly isolated from garlic (*Allium sativum* L.). It is known for its various pharmacological effects, including anti-microbial, anti-inflammatory, antioxidative, and anticancer properties [210]. It has been demonstrated that allicin inhibits the mouse colorectal tumorigenesis in the AOM/DSS mouse model. In HCT116 cells, it promotes apoptosis, suppresses proliferation, and reduces survival via activating the STAT3 pathway [211]. Other research indicated that allicin induces apoptosis in other CRC cell lines (HCT-116, LS174T, HT-29, and Caco-2) by regulating Nrf2 mediated cytochrome c releasing process [212]. The autophagy modulation property of allicin has been verified in different types of cells. Allicin-induced autophagy in SW1736 and HTh-7 thyroid cells caused the accumulation of ROS and the inhibition of AKT/mTOR which further alleviate the malignant development [213]. Allicin was able to induce p53-mediated autophagy in Hep G2 human liver cancer cells. In this study, it was observed that allicin inhibited the viability of Hep G2 cells by down-regulating the PI3K/mTOR signaling pathway associated proteins, and upregulating the AMPK/TSC2 and Beclin-1 expression level [214]. In addition, the allicin is reported to be capable of overcoming resistance to drug or radiation therapy in CRC treatment. Allicin improved the sensitivity of CRC cells to X-ray radiotherapy by a mechanism involving the inhibition of the NF-κB signaling pathway [215]. Administering allicin in combination with the chemotherapy agent 5-FU increases cancer cell death in both SK-MES-1 lung cancer and DLD-1 CRC cells. This synergistic effect could be a potential strategy for overcoming or preventing the development of drug resistance in CRC [216]. Though there is no specific autophagic modulation study of allicin in colorectal cancer cells until now, the tumor-inhibitory effect of allicin in colorectal cancer cells and its effectiveness in regulating autophagy in various cancer cells could make it a promising agent for colon cancer therapy.

**Table 1:** Natural compounds as modulators of autophagy for colorectal cancer prevention and therapy.

Compound	CRC Cell Line	Effect on Autophagy	Mechanism of Action	IC50	References
Curcumin	HT-29 HCT-15 HCT116	Activator	Inducing autophagy-related death by inhibiting the AKT/mTOR pathway or activating the ERK1/2 pathway Induction of autophagy via suppression of mTOR and activation of transcription factor EB (TFEB), and also cause cancer cell cycle accumulation at the G2/M phase	40 μM	[135,136,137,138,139,140,141,142,143,144,145,146,147,148,149,150]
Ursolic Acid	HCT-15	Activator	Inducing autophagy which is activated by the CaMKK-AMPK-mTOR pathway. Accumulation of LC3 and p62 levels and activation of caspase-independent cancer cell apoptosis through the JNK pathway	37.2 μM	[151,152,153,154,155,156,157,158]
Silibinin	SW480 LoVo HT-29 HCT116	Activator	Activation of autophagy via oxidative stress-mediated BNIP3-dependent manner or the mTOR inhibition manner Inhibition of cancer cell proliferation through extracellular signal-regulated kinase 1/2 (ERK1/2), AKT phosphorylation, and NF-κB inactivation manner	83 μM	[159,160,161,162,163,164,165,166]
Thymoquinone	LoVo HCT116	Inhibitor	Sensitize the chemotherapy by inhibiting NF-κB and MEK signaling Inhibition of the late stage of autophagy and cause the accumulation of LC3 and p62 levels	51.73 μM (HCT-116) 99.46 μM (HT29)	[167,168,169,170,171,172,173,174]
Dauricine Daurisoline	HCT116 HCT8 SW480 SW620	Inhibitor	Impairing autophagy at the autophagosome maturation stage due to p62 accumulation and the increased ratio of GFP-LC3/RFP-LC3 Suppression of colon cancer invasion via CaMKKβ-AMPK-mTOR signaling cascades pathway and NF-κB pathways	Both are >20 μM in HCT8 cell line	[175,176,177,178,179,180]
Bufalin	HT-29 Caco-2 HCT116	Activator	Inhibition of cancer cell proliferation by suppressing NF-κB and PI3K/AKT signaling pathways Activation of autophagy and apoptosis through inhibiting the phosphorylation of Akt and mTOR, while phosphorylated ERK1/2	12.823 ± 1.792 nM (HCT-116) 26.303 ± 2.498 nM (SW620)	[181,182,183,184,185]
Resveratrol	DLD1 HCT15 LoVo HCT116 HT-29 COLO 201	Activator	Activation of autophagic cell death through MALAT1 regulated WNT/β-catenin signaling pathway Induction of autophagy via ROS production and mTOR inhibition	170 μM	[186,187,188,189,190,191,192,193,194,195,196,197,198,199]
Sulforaphane	SW480, DLD1 HCT116 HT-29	Activator	Induction of autophagic cell death by modulating Wnt/β-catenin and ERK/Nrf2 signaling pathways	18.82 μM (DLD-1) 15.73 μM (HCT-116)	[200,201,202,203,204,205,206,207,208,209]
Allicin	HCT-116, LS174T HT-29 Caco-2	Activator	Activation of autophagy through ROS accumulation and the AKT/mTOR inhibition Inducing CRC cell apoptosis through regulating Nrf2 mediated cytochrome c and NF-κB releasing process	64.7 μM (SW620) 80 μM (HCT-116)	[210,211,212,213,214,215,216]

## 5. Clinical Study of Autophagy-Modulating Natural Products in Colon Cancer

The ability of natural products to inhibit tumor growth in animal models is crucial for assessing the druggability of phytochemicals. Growing evidence suggests that natural compounds that stimulate autophagy can effectively suppress tumor development in vivo [217,218]. The ultimate objective of the aforementioned studies is to identify naturally occurring compounds that induce autophagy and show promising therapeutic activity against colon cancer. Only a small number of clinical trials with autophagy-inducing natural compounds have been published to date. The precise mechanism of autophagy in the clinical management of cancer remains unclear. It is widely accepted that metastatic colorectal cancer (mCRC) is the final stage of CRC [219,220,221,222]. It has been proven in clinical trials that administering irinotecan to patients with advanced mCRC considerably increases survival rates and decreases the frequency of adverse effects [223]. Irinotecan has since been recognized as a standard cytotoxic agent, particularly in advanced mCRC patients who had failed in the initial phase of treatment with 5-FU. As a drug derived from a natural product, irinotecan remains a key component of combination chemotherapeutic agent for CRC, including XELIRI (capecitabine and irinotecan), IROX (irinotecan and oxaliplatin), and FOLFIRI (levoleucovorin, 5-FU, and irinotecan). Furthermore, several natural compounds are currently undergoing clinical testing for the treatment of CRC (Table 2). The information was obtained by searching the website https://clinicaltrials.gov using the keywords “colorectal cancer,” “intestinal adenomas” “natural compounds,” “curcumin,” “curcuminoids”, “silibinin,” “bufalin,” “dauricine” “daurisoline,” “allicin,” and “sulforaphane” (accessed on 7 March 2023).

In a study by Tanaka et al. [224] patients with colorectal adenomas were reported to show a significant reduction in both the size and number of colonic adenomas after 1 year of treatment with aged garlic extract, which contained the active allicin compound. A phase I clinical trial by Cheng et al. [225] was conducted to assess the safest dose of curcumin that can aid in preventing gastric intestinal metaplasia in patients. Injections of bufalin have been used clinically in some areas of China and demonstrated therapeutic efficacy in inhibiting tumor growth in cancer patients. Patients with different carcinomas treated with bufalin showed no dose-limiting toxicities and improved quality of life in a phase I pilot study [217].

Some natural products have the potential to be used in clinical settings; for example, resveratrol, which is present in many edible and medicinal plants, has been studied for its potential chemopreventive effects. Researchers found that intaking resveratrol in a phase I clinical trial with healthy volunteers did not result in any major adverse effects [226]. However, although resveratrol alone has been shown to have chemopreventive effects, it may interact with other medications due to its ability to control carcinogen-metabolizing enzymes in human models [227]. Clinical trials have shown that resveratrol positively impacts cancer patients when used alone or in conjunction with chemotherapeutic agents [191,228], and these findings have important implications for the clinical application of resveratrol in the management of cancer. Though no data have been made public that defines the connection between autophagy and the clinical action of these natural products, the effectiveness of some natural products in regulating autophagy demonstrated in in vitro and in vivo experiments and their long-term application in humans definitely holds promise to promote these natural products in clinical studies.

## 6. Discussion and Limitations

We have summarized the current reported natural products that inhibit CRC by regulating the autophagy pathway and the clinical trial these compounds are undergoing. However, there are some limitations and defects for these natural products which may hinder their further clinical drug development. Many phytomedicines and natural compounds possess various bioactivities, which also suggests that these compounds interact with multiple molecular proteins and this nonspecificity character may restrict their efficacy in clinical usage. For example, the latest research indicates that resveratrol directly interacts with more than 20 proteins, and also elaborates on the multiple beneficial effects and various bioactivities of resveratrol [229]. However, the anti-CRC effect of resveratrol may not simply be due to its autophagic modulation ability because of the nonspecificity of resveratrol. To minimize side effects and enhance the autophagic regulating impact of these reported natural compounds in CRC models, the chemical structure modification may benefit the precisely regulating autophagy and promote the clinical value of treating CRC [230]. Some researchers have modified the chemical structure of curcumin by deleting the β-diketone moiety to significantly enhance the autophagy activation effect and contribute to the direct binding with TFEB [231].

**Figure 3:**
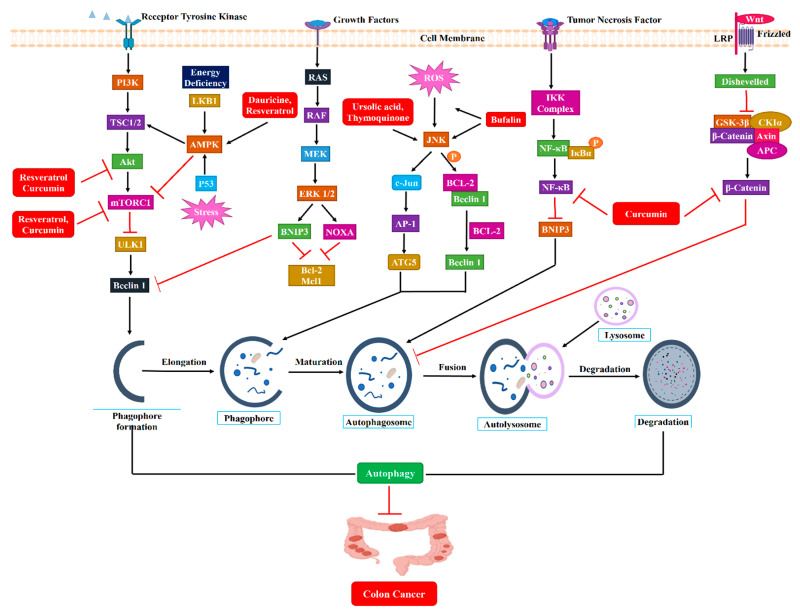
The relationship between the natural products targeting the major signaling pathways that modulate autophagy and colon cancer. Regulation of autophagy induction by the Unc-51-like kinase 1 (ULK1) complex is mediated by the PI3K/AKT pathway. mTOR is negatively regulated by LKB1/AMPK, while cytoplasmic p53 stimulates mTOR by blocking AMPK. Autophagy is also activated by the RAS/RAF/ERK pathway via Beclin-1 to facilitate nucleation. Expression of Atg5 regulates phagophore development, which is induced by reactive oxygen species, leads to the activation of c-Jun NH2-terminal kinases (JNKs). Bufalin could induce oxidative stress by activating JNK. JNK has the ability to phosphorylate Bcl-2 and liberate Beclin 1, thereby promoting autophagy. Alternately, JNK can stimulate the phosphorylation of c-jun; which promotes the generation of AP-1 that enhances the expression of numerous genes, including Beclin 1, Atg5, and Atg7. Beclin 1 and Atg 5 have the potential to induce autophagy. In contrast, the Wnt/β-catenin pathway activates and recruits β-catenin protein into the nucleus, thereby directly regulating autophagy. Without Wnt, a destruction complex degrades β-catenin in the canonical Wnt pathway, preventing cytoplasmic accumulation. The following proteins comprise this destruction complex: Axin, glycogen synthase kinase 3β (GSK3β), adenomatosis polyposis coli (APC), and casein kinase 1α (CK1α). On the other hand, Beclin 1 and some other autophagy-linked protein expression levels are increased by the IKKα/β and NF-κB pathways, inducing autophagy. In addition, NF-κB signaling can inhibit autophagy by overexpressing autophagy repressors such as Bcl-2/Xl and BNIP3. Natural products targeting the key signaling pathways that control autophagy in colon cancer. T-arrows represent inhibition, whereas arrows represent activation.

However, mounting evidence has validated the direct relationship between the specific autophagy modulators and the anti-CRC effect in recent decades. Currently, the widely accepted specific autophagy inhibitors, including chloroquine, hydroxychloroquine, bafilomycin A1, 3-MA, and wortmannin, have shown significant beneficial effects in the treatment of CRC models. Bafilomycin A1, the specific vacuolar V-ATPase inhibitor, has established a pronounced colon cancer inhibition effect by blocking the autolysosome fusion process of macroautophagy [232]. Wortmannin and 3-MA, the specific early-stage autophagy inhibitors that target the PI3KC3 complex, also exhibited therapeutic effects in colorectal cancer models [233]. The colorectal cancer therapeutic effects of late-stage autophagy inhibitors chloroquine and hydroxychloroquine have been widely demonstrated and have undergone several clinical trials, as summarized in the previous sections. The chemical structures of these specific autophagy modulators may allow us to employ a more precise and efficient chemical modification on our listed natural products, which may overcome the nonspecificity limitation and create a promising drug discovery pathway. The autophagic modulation pathway of these natural compounds has been illustrated in Figure 3.

Taken together, the nonspecificity and elusive precisely targeted pathway may limit the clinical value of these natural autophagy modulators in treating CRC, and further chemical modification may be needed to enhance their autophagic regulating effects and eliminate the effect side pathways, leading to more promising pharmacological developments for CRC.

## 7. Conclusions and Future Direction

Autophagy is a highly conserved process that sequesters misfolded proteins or damaged organelles into double-membrane vesicles and delivers them to lysosomes for degradation. Mounting evidence indicates that autophagy plays a pivotal role in supporting cancer cell survival as an adaptive mechanism under microenvironmental stress. However, since autophagy is regarded as a double-edged sword in tumorigenesis, either activation or inhibition of autophagy can suppress tumor growth remains controversial. On the one hand, since the autophagy level is continuously upregulated in various cancers, including CRC, autophagy will promote the self-recycling process to provide extra amino acids and ATP production under hypoxia or energy deprivation conditions to sustain cancer cell proliferation. Activating autophagy can be a potential therapeutic strategy since autophagy is also responsible for removing mutations or oncogenesis components. Moreover, continuous stimulation of autophagy by small molecules may trigger dysregulated homeostasis, excessive ER stress, or unfolded protein response, which will ultimately lead to apoptotic cell death with the accumulation of empty vacuoles. This autophagic cell death can thus induce cancer cell death and inhibit proliferation. Therefore, the autophagy modulator is regarded as a promising candidate for the treatment of CRC.

An increasing number of researchers have gradually accepted that inhibiting autophagy to overcome drug resistance (chemotherapy, radiotherapy, and immunotherapy) and inducing cancer cell death is a convincing road for CRC drug development. Both chloroquine and hydroxychloroquine, two potent autophagy inhibitors, have been demonstrated to possess potential value in cancer therapy and are being tested in phase II clinical trials for different types of cancers, including CRC. In this review, we also summarized the current information about ten well-known natural compounds that exhibit a CRC-suppressing effect via modulating autophagy. These natural compounds can be considered sources for CRC drug development because they have potent abilities to activate apoptosis and autophagy. Taken together, modulating autophagy is regarded as a potential, viable therapeutic target for CRC treatment. We hope these natural compounds can be used to bring progress in CRC drug development.

## Figures and Tables

**Table 2:** A list of natural compounds that have undergone clinical trials for the treatment of colorectal cancer.

Natural Compounds	Condition/Disease	Clinical Trial Number	Phase	Outcomes	Status
Curcumin and Avatin/FOLFIRI	Colorectal cancer	NCT02439385	2	Improves survival rates, safety, and tolerability	Completed
Curcumin and Irinotecan	Advanced colorectal cancer	NCT01859858	1	Evaluates the effect of curcumin on the pharmacokinetics of irinotecan	Completed
Curcumin C3 tablet	Colorectal cancer	NCT01333917	1	Measures gene expression, RNA levels, and apoptosis activity of curcumin	Completed
Oral complex C3 curcumin + chemotherapy	Colorectal cancer	NCT01490996	1	Evaluates the efficacy of curcumin in terms of disease response and survival	Completed
Curcumin	Colorectal cancer	NCT00027495	1	Determines the tolerable dose of curcumin that can aid in the prevention of colon cancer in healthy men and women.	Completed
Curcumin	Familial adenomatous polyposis	NCT00927485	Not applicable	Determines the safety and effectiveness of curcumin in reducing intestinal adenomas by counting the number of polyps in the duodenum, colon, and ileum.	Completed
Curcumin	Familial adenomatous polyposis	NCT00641147	2	Measures the efficacy and safety of curcumin in patients with familial adenomatous polyposis.	Completed
Curcuminoids	Colorectal cancer	NCT00027495	1	Evaluates the pharmacokinetics profile and the safest dose of curcumin for preventing colon cancer in healthy people.	Completed
Resveratrol	Colorectal cancer	NCT00433576	1	Inhibits tumor cell growth by inhibiting some of the enzymes required for cell proliferation.	Completed
SRT501 (Resveratrol)	Neoplasms, Colorectal cancer	NCT00920803	1	Determines the safety, tolerability, pharmacodynamics, and pharmacokinetic profile of SRT501 in blood and both normal and malignant metastatic tissue samples.	Completed
FOLFOX (Oxaliplatin, 5 Fluorouracil, Lederfolin)	Colon Cancer	NCT00646607	3	Evaluates the overall survival, toxicity, and adverse events	Completed

## Data Availability

Not applicable.
